# Differential posttraumatic stress disorder symptom cluster response to stellate ganglion block: secondary analysis of a randomized controlled trial

**DOI:** 10.1038/s41398-024-02926-8

**Published:** 2024-05-29

**Authors:** Shannon M. Blakey, Kristine L. Rae Olmsted, Shawn Hirsch, Kat Asman, Dennis Wallace, Murrey G. Olmsted, Russ Vandermaas-Peeler, Rhonda S. Karg, Bradford B. Walters

**Affiliations:** 1 RTI International, Research Triangle Park, NC USA; 2 Biostatistician-Retired, Durham, NC USA; 3 New Leaf Psychotherapy, Asheville, NC USA; 4 Independent researcher-Retired, Chapel Hill, NC USA

**Keywords:** Psychiatric disorders, Human behaviour

## Abstract

Empirically supported treatments for posttraumatic stress disorder (PTSD) exist, but research suggests these therapies are less effective, acceptable, and feasible to deliver to active duty service members (SMs) compared to civilians. Stellate ganglion block (SGB) procedure, in which a local anesthetic is injected around the cervical sympathetic chain or stellate ganglion to temporarily inhibit sympathetic nervous activity, is gaining popularity as an alternative PTSD treatment in military settings. However, it is unknown whether certain PTSD symptoms are more responsive to SGB than others. The current study involved a secondary analysis of data collected from a previous randomized controlled trial of SGB compared to sham (normal saline) injection (*N* = 113 SMs). PTSD symptoms were assessed via clinical interview and self-report at baseline and 8 weeks post-SGB or sham. Logistic regression analyses showed that the marked alterations in arousal and reactivity PTSD symptom cluster demonstrated the greatest symptom severity reductions after SGB, relative to sham. The reexperiencing cluster also showed pronounced response to SGB in clinician-rated but not self-reported outcomes. Post-hoc item-level analyses suggested that arousal and reactivity cluster findings were driven by reductions in hypervigilance, concentration difficulties, and sleep disturbance, whereas clinician-rated reexperiencing cluster findings were driven by reductions in physiological reactions to trauma cues, emotional reactions to trauma cues, and intrusions. Our findings align with a burgeoning literature positioning SGB as a potential novel or adjunctive PTSD treatment. Results could guide future hypothesis-driven research on mediators of therapeutic change during SGB for PTSD symptoms in SMs.

## Differential posttraumatic stress disorder symptom cluster response to stellate ganglion block: secondary analysis of a randomized controlled trial

Posttraumatic stress disorder (PTSD) is characterized by clinically significant distress and functional impairment related to directly experiencing, witnessing, or learning about a traumatic event. Outlined in the *Diagnostic and Statistical Manual for Mental Disorders* (DSM)*, Fifth Edition* [1], PTSD symptoms fall within four clusters: reexperiencing symptoms, avoidance of trauma-related stimuli, negative alterations in cognition or mood, and marked alterations in arousal and reactivity. Approximately 6% of the general U.S. population meets criteria for PTSD in their lifetime [2], but rates are as high as 20% among U.S. service members (SMs) and veterans [3]. This civilian–military discrepancy in lifetime PTSD prevalence may be explained by elevated occupational risk for trauma exposure during military service in addition to potential pre-military or non-military trauma exposure [4, 5].

Fortunately, people with PTSD have multiple treatment options. Several clinical practice guidelines converge to recognize trauma-focused therapy (TFT) as the first-line treatment for PTSD [6]. The two most studied and empirically supported TFTs are Prolonged Exposure [7] and Cognitive Processing Therapy [8]. These therapies are considered trauma-focused because they involve systematically confronting trauma-related thoughts and stimuli. TFTs have demonstrated large effect sizes from pre- to post-treatment in meta-analytic studies [9]. However, clinical trials conducted in both highly controlled [10] and community [11] settings suggest that the therapeutic effects of TFTs are attenuated for SMs and veterans, relative to civilians. In fact, a meta-analysis of randomized controlled trials (RCTs) of exposure-based TFTs delivered to SMs or veterans found that effects were only small to medium in magnitude [12].

Although the Department of Defense (DoD) strongly recommends TFTs for SMs with PTSD [13], there are several barriers to disseminating TFTs to active duty military personnel. Providers at military treatment facilities (MTF) often carry large caseloads that preclude scheduling 12 to 16 consecutive, weekly, 60- to 90-minute sessions, as TFTs are meant to be delivered [14]. Orders to deploy, participate in off-site training, or change duty stations can also disrupt SMs’ care, and consequently clinic flow. TFTs can be efficacious when delivered in massed format (i.e., same overall treatment time but condensed to span fewer weeks) or variable length [15, 16], but these alternative delivery schedules tested in research settings may still be impractical for many MTF providers. Additionally, feasibility challenges to implementing TFTs are compounded by pervasive stigma in the military against seeking mental health care more broadly [17–19]. Even generally adaptive military cultural values, such as self-reliance, can interfere with a SM’s full engagement with TFT [20]. In a large sample of soldiers with a diagnosed mental health disorder, the most common attitudinal barrier to initiating (77.0%) or completing (52.5%) treatment was a preference to “handle problems on one’s own” [21].

A third barrier to successful dissemination of TFTs to SMs regards the trauma focus. Specifically, some MTF [14] and non-military [22] clinicians are hesitant to deliver TFTs because of these treatments’ emphasis on confronting distressing trauma-related stimuli. Although such concerns are often mistaken and can be addressed through corrective information [23], there is evidence that some patients find TFTs difficult to tolerate. In a large Department of Veterans Affairs PTSD specialty clinic, 38.5% of veterans who initiated TFT dropped out, and more than one quarter of those did so before their third TFT session [24]. This finding is clinically meaningful, as the third session is when patients begin engaging in imaginal exposure to their trauma memory in Prolonged Exposure or challenging their trauma-related beliefs in Cognitive Processing Therapy. Taken together, although steps should be taken to make TFTs more feasible, acceptable, and effective in military settings, there is also a need to identify alternative treatment options for SMs with PTSD who cannot or will not complete TFTs.

One adjunctive approach gaining popularity is stellate ganglion block (SGB) [25]. During an SGB procedure, a local anesthetic is injected around the cervical sympathetic chain or stellate ganglion (a cluster of nerves and nerve fibers located at the base of the neck) to temporarily “block” the stellate ganglion’s function and inhibit sympathetic (“fight-or-flight”) nervous activity. SGB has been used for more than a century to treat sympathetically mediated conditions such as complex regional pain syndrome, migraines, facial pain, upper extremity pain, and hot flashes [26]. The application of SGB to treat PTSD symptoms, however, is relatively recent. Building on individual case studies describing patients whose PTSD symptoms abated after SGB, Mulvaney and colleagues [27] published a case series of 166 active duty SMs who received SGB for PTSD. These authors reported statistically significant reductions in PTSD symptoms after the procedure, which were maintained out to 3-month follow-up. Causality could not be determined, however, because there was no control group. Two RCTs of SGB have been published to date. Hanling and colleagues [28] failed to detect significant differences between SGB and sham injection on PTSD outcomes in a sample of 42 SMs, but these null conclusions are tenuous given the trial’s methodological limitations (see Lipov, 2014) [29]. In a larger RCT of 113 SMs, Rae Olmsted and colleagues [30] found that SGB led to a significantly greater reduction in PTSD symptoms compared to sham injection, with a medium effect size at 8 weeks post-SGB. Controlled research on SGB for PTSD, while burgeoning, is still nascent. It is therefore important to consider the potential for publication bias as an explanation for promising limited findings. As some assurance, a recent evidence brief synthesizing research on SGB used to treat PTSD symptoms incorporated “gray literature searching” and concluded that SGB might be an effective approach that should continue to be rigorously evaluated [26].

The “off-label” use of SGB for PTSD is likely to expand across MTFs as more providers and SMs learn about this procedure [25]. Although there are certain risks involved with an SGB procedure, ranging from minor (e.g., cough, hoarseness, light-headedness) to severe (e.g., seizure, brachial plexus block, subdural block/intraspinal blockade) [31], SGB is considered acceptably (if not completely) safe when performed by competent practitioners [26], and it is well-tolerated and accepted by SMs [32]. One MTF quality assurance project found that SMs (*N* = 110) who received SGB for their PTSD symptoms experienced only mild or no discomfort during the procedure, perceived few side effects of SGB, endorsed high satisfaction with SGB, and said they would recommend SGB to a friend [32]. From an MTF personnel perspective, the relative advantages of SGB over established PTSD treatments include near-immediate symptom relief (the anesthetic is fast-acting), less provider burden (the procedure itself takes about 5 min, with the entire visit lasting no more than 30 min), and potentially better patient compliance (though this remains an empirical question).

Despite evidence that SGB can be effective for SMs with PTSD, the mechanism(s) by which SGB ameliorates PTSD symptoms is unknown. Purported explanations for SGB’s therapeutic effects include neural inhibition in the sphere of innervation, reduced brain norepinephrine via reduced nerve growth factor, better regulated amygdala activity, biologic sedation, and melatonin biosynthesis [33–35]. Based on these assumptions, it stands to reason that certain PTSD symptoms may be more responsive to SGB than others. Specifically, inhibited sympathetic nervous system activity after an SGB procedure might *especially* relieve symptoms of hyperarousal and reactivity associated with the fight-or-flight response. The arousal and reactivity PTSD cluster includes symptoms of irritability and angry outbursts, reckless or self-destructive behavior, hypervigilance, exaggerated startle response, concentration difficulties, and sleep disturbance [1]. One reexperiencing cluster symptom—having strong physiological reactions to internal or external trauma-related stimuli—might also markedly improve due to SGB’s presumed downregulating effects on sympathetic nervous system activity.

Supporting empirical evidence is scant, but one study found that the PTSD symptoms associated with the greatest improvement from pre-SGB to 2-month follow-up were irritability, concentration difficulties, and sleep disturbance [36]. These findings are limited, however, by the study’s small sample (*N* = 30) and lack of control group. Assessing PTSD symptoms according to a previous edition of the DSM [37] (DSM-IV-TR) may further limit future applicability of these findings because PTSD diagnostic criteria underwent significant changes from DSM-IV-TR to DSM-5, including an increase from 17 to 20 total symptoms, removal of one symptom, and rearrangement of symptoms from three clusters to four. Extant RCTs using DSM-5 PTSD assessments have not examined PTSD cluster-level improvement after SGB, as these trials reported *total* PTSD symptom severity scores as their outcome [28, 30].

The current study aimed to build on existing work and examine whether certain PTSD symptom clusters are especially responsive to SGB. Based on previous related research [36] and the conceptual relevance of physiological PTSD symptoms to SGB’s purported mechanisms of action, we expected that the marked alterations in arousal and reactivity cluster (i.e., DSM-5 PTSD Cluster E) would show the greatest therapeutic change from baseline to 8 weeks post-SGB procedure. We also planned to conduct exploratory analyses of symptom-level change within the PTSD cluster(s) showing the greatest response to SGB. To maximize the reliability of our findings, we conducted a secondary analysis of a completed RCT of SGB for SMs with PTSD symptoms [30]. Because the current secondary analysis aims were not part of the original RCT study design, we consider our current aims to be hypothesis *generating* rather than hypothesis testing.

## Materials and Methods

### Participants and Procedure

Data were collected from 113 SMs recruited at 3 U.S. MTFs as part of a larger RCT (ClinicalTrials.gov Identifier NCT03077919) [30]. SMs were eligible to participate in the RCT if they were serving on active duty status, had been on a stable dose of psychotropic medications for at least 3 months (if taking psychotropic medications), and scored at least a 32 on the PTSD Checklist–Civilian Version for DSM-IV (PCL-C) [38]. The PCL-C was used to determine participant eligibility because recommended screening cut-points had not yet been determined for the then-recently published PTSD Checklist for DSM-5 (PCL-5) [39], whereas screening cut-points were available for the PCL-C [40]. SMs were excluded from participating if they had previously received SGB; had a lifetime diagnosis of bipolar disorder, a psychotic disorder, or a personality disorder; endorsed moderate to severe past-30-day substance use disorder symptoms; had a moderate or severe traumatic brain injury; endorsed past-2-month suicidal ideation; or were determined by the provider who administered the SGB procedure to be at risk for injury or adverse outcomes (such as undergoing a medical board evaluation due to concerns about fitness for duty) related to any health condition. The sample was mostly (88.5%) male and had a mean age of 37.3 years (*SD* = 6.7). Most (*n* = 91; 80.5%) participants met the full diagnostic criteria for PTSD according to DSM-5.

After providing written informed consent, participants were randomized 2:1 to SGB (7 to 10 mL of ropivacaine, 0.5%) or sham (1 to 2 mL of normal saline) using a central stratified block assignment scheme. Blinded random allocation was stratified by study site. Immediately prior to the procedure, the treating provider was informed of the participant’s group assignment. Identical SGB or sham procedures were administered at baseline and 2 weeks later (i.e., weeks 0 and 2). Participants were compensated if they completed study assessments during their off-duty time in accordance with DoD policies regarding paying DoD employees for research participation [41]. The secondary analyses reported here were approved by one of the study site Institutional Review Boards, with the two other study sites and the study data and coordinating center IRBs deferring to the single study site IRB [30]. Approval was also granted by the U.S. Army Medical Research and Development Command’s Human Research Protection Office. Additional study procedure information, including study recruitment, enrollment, and visit completion rates, are described elsewhere [30].

## Measures

### Clinician-Administered PTSD Scale for DSM-5

(CAPS-5) [42]. The CAPS-5 was administered via telephone by trained clinical psychologists at baseline and at 8 weeks after the baseline SGB procedure (i.e., week 8). Considered the gold standard PTSD assessment instrument [43], the CAPS-5 assesses the past-month frequency and intensity of each PTSD symptom on a score of 0 (*absent*) to 4 (*extreme/incapacitating*). The 20 individual item severity scores are summed to yield a total symptom severity score (TSSS) ranging from 0 to 80, with higher scores indicating more severe PTSD symptoms. PTSD symptom cluster scores can also be calculated by summing the relevant item scores (reexperiencing cluster [5 items] scores range from 0 to 20; avoidance cluster [2 items] scores range from 0 to 8; cognition and mood cluster [7 items] scores range from 0 to 28; arousal and reactivity cluster [6 items] scores range from 0 to 24).

### PTSD Checklist for DSM-5

(PCL-5) [39]. Participants completed the PCL-5 at baseline and week 8. Participants self-report the extent to which they were bothered by each of the 20 PTSD symptoms in the past month using a scale of 0 (*not at all*) to 4 (*extremely*). Like the CAPS-5, PCL-5 item scores are summed to yield a self-reported TSSS ranging from 0 to 80, as well as cluster severity scores (ranges vary by cluster identically to the CAPS-5). The PCL-5 has demonstrated strong psychometric properties in undergraduate and veteran samples [44, 45].

## Data analytic plan

Because the current study involved a secondary analysis of a previously completed RCT and was focused on hypothesis generation, no formal power calculations were performed to determine the sample size. Due to the exploratory nature of this secondary analysis study, all comparisons described below were descriptive in nature; no tests of statistical significance were performed. All analyses were conducted using SAS version 9.4. Although analytic code is publicly unavailable, the corresponding author may be able to share analysis code upon request.

First, we calculated the proportion of SMs who endorsed sufficient symptoms in each PTSD cluster to support a formal diagnosis of PTSD at both baseline and week 8. As specified in the DSM-5 [1], we used the following symptom cluster-level diagnostic threshold criteria: at least one re-experiencing symptom (Cluster B), at least one avoidance symptom (Cluster C), at least two cognition and mood symptoms (Cluster D), and at least two arousal and reactivity symptoms (Cluster E). These analyses were conducted separately by cluster and by measure (i.e., CAPS-5 and PCL-5), adhering to scoring conventions whereby item-level severity scores of 2 or higher were considered clinically significant (i.e., “counting” toward a cluster threshold and PTSD diagnosis) [39, 42].

Next, we conducted logistic regression models (adjusted analyses) of each PTSD symptom cluster separately for the CAPS-5 and PCL-5, using *not* meeting the PTSD cluster-level clinical threshold at week 8 as the outcome and study site and treatment group (i.e., SGB or sham) as predictors. As the objective of this secondary analysis was to examine which PTSD clusters were most responsive to SGB, each model was subset to only include participants that *met* the cluster-level clinical threshold at baseline.

Finally, we performed ad-hoc exploratory analyses on the two PTSD clusters that the above-described descriptive comparisons suggested were most responsive to SGB relative to sham. For these analyses, we calculated the mean *symptom-*level change from baseline to week 8 separately for those two PTSD symptom clusters, independently for the CAPS-5 and PCL-5.

## Results

### Descriptive analyses

For each symptom cluster and outcome measure, Table 1 presents the proportion of SMs who (a) always met the cluster-level clinical threshold (i.e., met the clinical threshold at baseline and week 8), (b) remitted from the cluster-level clinical threshold (i.e., met the clinical threshold at baseline but not week 8), (c) emerged to meet the cluster-level clinical threshold (i.e., did not meet the clinical threshold at baseline but did at week 8), or (d) never met the cluster-level clinical threshold (i.e., did not meet the clinical threshold at baseline or week 8). For CAPS-5, the largest differences in the proportion of SMs who remitted from cluster-level clinical threshold status were observed in the reexperiencing cluster (27.1% for SGB vs. 13.2% for sham) and the arousal and reactivity cluster (18.6% for SGB vs. 5.3% for sham). For PCL-5, the largest difference in the proportion of SMs who remitted from cluster-level clinical threshold status was observed in the arousal and reactivity cluster (32.4% for SGB vs. 10.8% for sham).

**Table 1:** Change in PTSD symptom cluster diagnostic threshold status^1^ from baseline to week 8.

PTSD Symptom Measure and Symptom Cluster	Treatment Group *n* (%)	
	SGB	Sham
**CAPS-5 Cluster B (Reexperiencing)**		
Always Met	48 (68.6%)	32 (84.2%)
Remitted	19 (27.1%)	5 (13.2%)
Emergent	1 (1.4%)	0 (0.0%)
Never Met	2 (2.9%)	1 (2.6%)
**CAPS-5 Cluster C (Avoidance)**		
Always Met	46 (65.7%)	27 (71.1%)
Remitted	17 (24.3%)	8 (21.1%)
Emergent	1 (1.4%)	1 (2.6%)
Never Met	6 (8.6%)	2 (5.3%)
**CAPS-5 Cluster D (Negative Cognition/Mood)**		
Always Met	50 (71.4%)	28 (73.7%)
Remitted	19 (27.1%)	6 (15.8%)
Emergent	1 (1.4%)	1 (2.6%)
Never Met	0 (0.0%)	3 (7.9%)
**CAPS-5 Cluster E (Arousal/Reactivity)**		
Always Met	55 (78.6%)	36 (94.7%)
Remitted	13 (18.6%)	2 (5.3%)
Emergent	0 (0.0%)	0 (0.0%)
Never Met	2 (2.9%)	0 (0.0%)
**PCL-5 Cluster B (Reexperiencing)**		
Always Met	35 (49.3%)	25 (67.6%)
Remitted	29 (40.8%)	10 (27.0%)
Emergent	1 (1.4%)	0 (0.0%)
Never Met	6 (8.5%)	2 (5.4%)
**PCL-5 Cluster C (Avoidance)**		
Always Met	34 (47.9%)	22 (59.5%)
Remitted	24 (33.8%)	7 (18.9%)
Emergent	1 (1.4%)	2 (5.4%)
Never Met	12 (16.9%)	6 (16.2%)
**PCL-5 Cluster D (Negative Cognition/Mood)**		
Always Met	38 (53.5%)	21 (56.8%)
Remitted	25 (35.2%)	8 (21.6%)
Emergent	4 (5.6%)	2 (5.4%)
Never Met	4 (5.6%)	6 (16.2%)
**PCL-5 Cluster E (Arousal/Reactivity)**		
Always Met	44 (62.0%)	32 (86.5%)
Remitted	23 (32.4%)	4 (10.8%)
Emergent	1 (1.4%)	1 (2.7%)
Never Met	3 (4.2%)	0 (0.0%)

*PTSD* Posttraumatic stress disorder, *CAPS-5* Clinician-Administered PTSD Scale for DSM-5, *PCL-5* PTSD Checklist for DSM-5, *SGB* Stellate ganglion block.

^1^Diagnostic threshold status refers to whether sufficient symptoms were met in each PTSD symptom cluster to satisfy DSM-5 diagnostic criteria for that cluster. Always met = Diagnostic threshold met for that cluster at baseline and week 8; Remitted = Diagnostic threshold met for that cluster at baseline but not at week 8; Emergent = Diagnostic threshold not met for that cluster at baseline but met at week 8; Never met = Diagnostic threshold for that cluster was not met at either baseline or week 8.

### Logistic regression analyses

Results from the logistic regression models are presented in Table 2. Among SMs meeting the cluster-level clinical threshold at baseline, the SGB group had higher odds of *not* meeting the clinical threshold at week 8, compared to the sham injection group, across all PTSD symptom clusters and outcome measures. CAPS-5 analyses for the arousal and reactivity cluster produced the largest odds ratio in favor of SGB, *OR* = 4.58, 95% CI [0.96–21.90], followed by CAPS-5 analyses for the reexperiencing cluster, *OR* = 2.49, 95% CI [0.84–7.39]. For PCL-5, the arousal and reactivity cluster analyses produced the largest odds ratio in favor of SGB, *OR* = 4.25, 95% CI [1.33–13.54].

**Table 2:** Logistic regression models of not meeting the PTSD cluster clinical threshold at week 8.

Measure	PTSD Symptom Cluster	SGB vs. Sham Odds Ratio [95% CI]
CAPS-5	Cluster B (Reexperiencing)	2.49 [0.84, 7.39]
	Cluster C (Avoidance)	1.20 [0.45, 3.19]
	Cluster D (Negative cognition/mood)	1.81 [0.64, 5.11]
	Cluster E (Arousal/reactivity)	4.58 [0.96, 21.90]
PCL-5	Cluster B (Reexperiencing)	2.13 [0.87, 5.19]
	Cluster C (Avoidance)	2.20 [0.80, 6.04]
	Cluster D (Negative cognition/mood)	1.72 [0.65, 4.51]
	Cluster E (Arousal/reactivity)	4.25 [1.33, 13.54]

In CAPS-5 analyses, 108 participants met the cluster B clinical threshold at baseline (70 SGB and 38 Sham), 102 participants met for cluster C at baseline (66 SGB and 36 Sham), 106 participants met for cluster D at baseline (71 SGB and 35 Sham), and 111 participants met for cluster E at baseline (72 SGB and 39 Sham). In PCL-5 analyses,102 participants met the cluster B clinical threshold at baseline (66 SGB and 36 Sham), 90 participants met for cluster C at baseline (60 SGB and 30 Sham), 94 participants met for cluster D at baseline (64 SGB and 30 Sham), and 108 participants met forcluster E at baseline (70 SGB and 38 Sham).

*CI* confidence interval.

### Symptom-level change (Post-Hoc) analyses

**Fig. 1:**
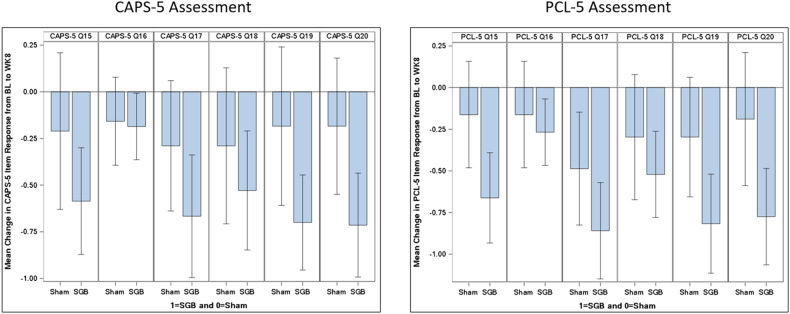
Mean Change in Item-Level Scores for CAPS-5 and PCL-5 Cluster E (Arousal and Reactivity) Symptoms, by Treatment Condition. Note. Bl = Baseline assessment; WK8 = Week 8 assessment; Q15 = Irritable or angry behavior; Q16 = Risk-taking behavior; Q17 = Hypervigilance; Q18 = Exaggerated startle; Q19 = Concentration difficulties; Q20 = Sleep disturbance.

**Fig. 2:**
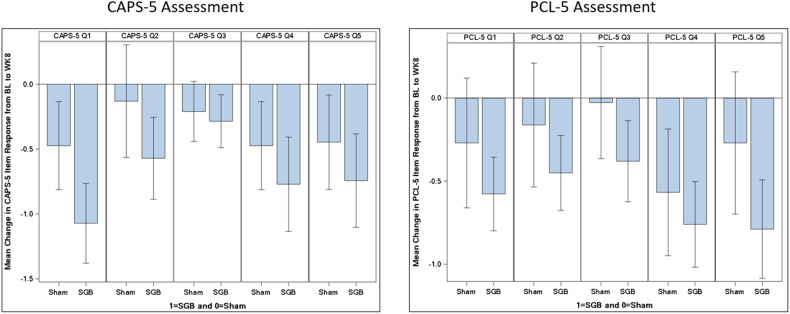
Mean Change in Item-Level Scores for CAPS-5 and PCL-5 Cluster B (Reexperiencing) Symptoms, by Treatment Condition. Q1 = Intrusive memories; Q2 = Nightmares; Q3 = Flashbacks; Q4 = Emotional reactions to traumacues; Q5 = Physiological reactions to trauma cues.

Mean changes in CAPS-5 item-level scores for the symptoms in the arousal and reactivity cluster (6 items) and the reexperiencing cluster (5 items) are illustrated in Figs. 1 and 2, respectively. Among SMs who received SGB, the three arousal and reactivity cluster symptoms that demonstrated the largest improvement from baseline to week 8 were hypervigilance (item 17; decrease of 0.67 points), concentration difficulties (item 19; decrease of 0.70 points), and sleep disturbance (item 20; decrease of 0.71 points). The only arousal and reactivity symptom with almost no change from baseline to week 8 was risk-taking behavior (item 16; decrease of 0.19 points). Regarding the reexperiencing cluster, the symptom that demonstrated the largest improvement from baseline to week 8 was intrusive memories (item 1; decrease of 1.07 points). Of note, this symptom also had the largest mean change difference between the SGB and sham groups (mean group difference of 0.50 points). The two other reexperiencing cluster symptoms showing relatively greater improvements from baseline to week 8 were emotional reactions to trauma cues (item 4; decrease of 0.77 points) and physiological reactions to trauma cues (item 5; decrease of 0.74 points).

Mean changes in PCL-5 item-level scores for the symptoms in the arousal and reactivity cluster and the reexperiencing cluster are shown in Figs. 1 and 2, respectively. Among SMs in the SGB group, the two arousal and reactivity cluster symptoms that demonstrated the largest change from baseline to week 8 were hypervigilance (item 17; decrease of 0.86 points) and concentration difficulties (item 19; decrease of 0.82 points). The arousal and reactivity symptom with the largest mean change *difference* between the SGB and sham groups was sleep disturbance (item 20; mean group difference of 0.59 points). For the reexperiencing cluster, the two symptoms that demonstrated the largest improvement from baseline to week 8 were physiological reactions to trauma cues (item 5; decrease of 0.79 points) and emotional reactions to trauma cues (item 4; decrease of 0.76 points). The reexperiencing cluster symptom with the largest mean change difference between the SGB and sham groups was marked physiological reactions to trauma cues (item 5; mean group difference of 0.52 points).

## Discussion

Despite the development of effective TFTs for people with PTSD, research evidence indicates these approaches are less acceptable, effective, and feasible to deliver to active duty SMs compared to civilian adults [12, 14]. SGB, a brief medical procedure that has long been used to treat pain and related conditions, has shown promise as a potential novel PTSD treatment [30]. In light of SGB’s growing popularity among SMs and the military medical community [25, 32], research aimed at explicating differential PTSD symptom response to this procedure represents an important step toward understanding the clinical presentations for which SGB might be most effective.

In our exploratory secondary analysis of data collected as part of an RCT of 113 SMs screening positive for PTSD [30], we found that the PTSD symptom clusters demonstrating the greatest symptom severity reductions after SGB, relative to sham injection, were the marked alterations in arousal and reactivity cluster (in CAPS-5 and PCL-5 analyses) and the reexperiencing cluster (in CAPS-5 analyses only). Item-level analyses suggested that arousal and reactivity cluster findings were driven by reductions in hypervigilance, concentration difficulties, and sleep disturbance, whereas reexperiencing cluster findings were driven by reductions in physiological reactions to trauma cues, emotional reactions to trauma cues, and intrusions (for CAPS-5 analyses only). Our findings are generally consistent with those reported in a smaller case series that analyzed DSM-IV-TR PTSD cluster- and symptom-level severity change after SGB [36].

Although this study did not assess mediators of therapeutic change, the relevant literature offers potential explanations that could guide future hypothesis-driven research. For example, SGB might downregulate sympathetic overactivity [26, 46], resulting in immediate and noticeable reductions in physiological, cognitive, and affective fight-or-flight symptoms. Such reductions could lead to secondary improvements in other PTSD symptoms as well; indeed, a recent observational study found that adult trauma survivors (*N* = 285) receiving SGB reported clinically meaningful reductions in anxiety symptoms, which were maintained out to 1 month post-SGB [47]. Alternatively, adjacent research in the anxiety disorder literature emphasizes the relevance of *ex-consequentia reasoning* and *anxiety sensitivity*, both of which could help explain SGB’s effectiveness in treating PTSD symptoms. Ex-consequentia reasoning is the phenomenon whereby individuals with elevated anxiety infer threat from their subjective anxious experiences (i.e., “if I’m anxious, there must be danger”) [48]. Experimental studies conducted in civilian and veteran samples suggest that ex-consequentia reasoning contributes to the development and persistence of PTSD [49, 50]. Similarly, anxiety sensitivity refers to the emotional distress and physiological activation that arises from mistaken beliefs about the meaning or significance of anxious arousal (i.e., “the fear of fear”) [51]. Given research highlighting a reciprocal relationship between PTSD symptoms and anxiety sensitivity [52, 53], it is possible that reducing the frequency and/or intensity of fight-or-flight symptoms via SGB could allow SMs to make more balanced, objective interpretations of their situational threat, thereby averting a “vicious cycle” of self-exacerbating PTSD symptoms.

One interesting observation from our study was the slightly different pattern of results as a function of whether PTSD symptom severity was measured via clinician rating (i.e., CAPS-5) or self-report (i.e., PCL-5). Specifically, sleep disturbance and intrusive memories showed larger improvements according to the CAPS-5, but not the PCL-5. Although CAPS-5 and PCL-5 data are not contradictory, our findings nevertheless underscore important nuances in how PTSD symptoms are experienced by SMs and perceived by trained evaluators. Although some degree of discordance between patient- and clinician-report would be expected [43, 54], other research has suggested that clinician-rated and self-reported PTSD severity measures capture overlapping but slightly different latent constructs [55]. Taken together, our findings suggest that future trials of SGB for SMs with PTSD should include both clinician-rated and self-report outcomes to comprehensively evaluate SGB’s treatment effects.

This study’s findings point to several potential future directions. First, although tested as a stand-alone intervention in the original RCT [30], SGB could potentially be used as an adjunct treatment to first-line TFTs. As our analyses suggested that reexperiencing and arousal/reactivity symptoms were the PTSD clusters most responsive to SGB, this procedure might help SMs to better engage in elements of TFTs, which can be challenging for some patients to complete [56, 57]. By alleviating anxiety-related physiological symptoms, SGB could potentially reduce dropout and increase compliance and engagement with TFTs, as has been suggested for other anxiety-related disorders [58]. Indeed, prior studies of civilians and veterans with PTSD showed that hyperarousal symptoms were associated with weaker neural habituation to aversive stimuli [59] and poorer response to Cognitive Processing Therapy [60]. As one pilot study combining SGB with Prolonged Exposure to treat combat-related PTSD (*N* = 12) reported clinically significant change without evidence of more adverse events compared to standalone SGB and PE trials [61], additional studies comparing the effects of SGB delivered alone or delivered alongside TFTs are warranted. Indeed, two clinical trials pairing SGB and TFT are ongoing and will hopefully inform how to best combine or sequence these approaches in the future.^1^

Second, SGB could be used in a stepped model of care [62]. For instance, SMs with sub-threshold PTSD or who have limited availability or interest in completing TFTs could be offered SGB as a lower-intensity, lower-burden treatment option. SMs who continue to experience clinically significant symptoms could then be offered future TFT or other clinically indicated mental health care once they have greater availability or interest. Similarly, when patient demand for PTSD treatment exceeds clinical supply, providers could consider the potential risks and benefits of referring SMs for an SGB procedure as an intermediary treatment option, rather than placing them on TFT waitlists without any active intervention. Importantly, the potential health risks of the SGB procedure—including the severity and likelihood of those risks—should be clearly communicated to SMs considering this treatment option. These documented risks include (from most to least common) [31] hoarseness, hematoma, light-headedness, blood aspiration, hypertension, brachial plexus block, dysphagia, cough, intrathoracic bleeding, subdural block/intraspinal blockade, seizures, transient locked-in syndrome, dyspnea and respiratory depression, migraine and headaches, persistent ptosis, pneumothorax, infection, bilateral sympathetic blockade, decreased contralateral blood flow, contralateral Horner’s syndrome, bradycardia, visual hallucinations, bloodshot conjunctiva, myoclonus, arm numbness, reading difficulty, bilateral Horner’s syndrome, lower limb edema, transient global amnesia, intrajugular vein thrombosis, allergic reaction, hemidiaphragmatic paralysis, dural puncture, hemomediastinum, sinus arrest due to vasovagal reflux, transient neurologic injury, laryngeal mask airway puncture, and asystolic cardiac arrest. Although advances in procedure techniques and technologies have improved visualization of the injection area during the procedure, serious complications from inaccurate needle placement can happen [26, 31].

Third, if SGB continues to demonstrate treatment effectiveness for PTSD symptoms, it may eventually be recommended as an empirically supported PTSD treatment option. Given the limited body of research evaluating SGB used to treat PTSD symptoms, however, additional controlled studies are needed. At the time of writing, the World Health Organization’s International Clinical Trials Registry Platform indicates there are 11 ongoing clinical trials of SGB for the treatment of PTSD in addition to three already completed SGB trials [28, 30, 61]. Future PTSD treatment evidence syntheses and clinical practice guidelines precipitated by these and future SGB studies should consider not only published trial findings, but also the potential for publication bias that may result from the tendency for studies with null findings to not be submitted or accepted for publication in scientific outlets (i.e., the “file drawer” phenomenon). Future research will also need to identify and validate the mechanism(s) of action for SGB in alleviating symptoms of PTSD. SGB appears to relieve PTSD symptoms, although it is not yet known *how* SGB leads to the specific symptom reductions observed in the literature. Improved understanding of the mechanisms underlying SGB’s therapeutic effects could lead to enhanced patient care by improving the safety, effectiveness, and efficiency of PTSD treatment as well as determining the best course of patient care [63, 64]. Future research should be conducted to determine if the findings observed in these analyses are stable across other samples and settings. Given the medical nature of the procedure, SGB could potentially be more acceptable to SMs who endorse stigma or other perceived barriers to seeking mental health care. Future studies should therefore also empirically test whether SGB is more palatable or accessible to SMs with PTSD, compared to psychological treatments.

The present study is limited by several factors. First, these analyses were exploratory in nature, and the original study from which they were drawn was not powered a priori to test symptom cluster-level effects. Additional studies using larger samples could test hypotheses regarding differential PTSD symptom cluster response to SGB, compared to inert or extant active controls (e.g., TFTs). Another limitation of post-hoc secondary analyses is the increased risk of a type I error inherently associated with testing hypotheses beyond those included in the original trial design and the subsequent potential for generating biased inferences. We attempted to mitigate this risk by reporting effect sizes and 95% confidence intervals rather than reporting the results as *p*-values from formal hypothesis tests. As previously stipulated, these analyses and associated results should be framed as hypothesis-generating rather than as evidence for the basis of formal inference, especially given how few trials of SGB for PTSD have been conducted. Systematic reviews and meta-analyses would also make an important contribution to the literature once this area of research further develops.

Second, this study did not measure biomarkers of sympathetic nervous system activity, which could bolster findings by indicating whether observed differences in symptom clusters have biological correlates. Future research should incorporate objective measures as part of multimodal assessment to investigate the potential mediators of symptom change after SGB. Third, because the original RCT required SMs to satisfy several eligibility criteria to participate, our findings may not generalize to the entire population of SMs with PTSD symptoms or to civilians with PTSD symptoms. These limitations notwithstanding, our findings build on previous research and point to some promising new directions in the study and treatment of PTSD symptoms using SGB.

## Data Availability

The data analyzed in this study are not openly available but may be available from the original clinical trial (NCT03077919) Principal Investigator upon reasonable request. Data are stored and managed at RTI International.

## Footnotes

1 National Library of Medicine. (2023, June 8). *Combining stellate ganglion block with prolonged exposure for PTSD* (Identifier NCT05889741). https://clinicaltrials.gov/study/NCT05889741. National Library of Medicine. (2022, November 21). *Enhancing cognitive processing therapy for posttraumatic stress disorder via stellate ganglion block treatment: A pilot trial* (Identifier NCT05534126). https://clinicaltrials.gov/study/NCT05534126.
