# Correction: Differential posttraumatic stress disorder symptom cluster response to stellate ganglion block: secondary analysis of a randomized controlled trial

**DOI:** 10.1038/s41398-024-02983-z

**Published:** 2024-06-25

**Authors:** Shannon M. Blakey, Kristine L. Rae Olmsted, Shawn Hirsch, Kat Asman, Dennis Wallace, Murrey G. Olmsted, Russ Vandermaas-Peeler, Rhonda S. Karg, Bradford B. Walters

**Affiliations:** 1 RTI International, Research Triangle Park, NC USA; 2 Biostatistician-Retired, Durham, NC USA; 3 New Leaf Psychotherapy, Asheville, NC USA; 4 Independent researcher-Retired, Chapel Hill, NC USA

**Keywords:** Psychiatric disorders, Human behaviour

Correction to: *Translational Psychiatry* 10.1038/s41398-024-02926-8, published online 29 May 2024

In this article, the affiliation details for Rhonda S. Karg were incorrectly given as, ‘New Leaf Psychotherapy, Ashville, NC, USA.’ but should have been, ‘New Leaf Psychotherapy, Asheville, NC, USA.’

The original article has been corrected.

